# Measures of Ethics and Social Responsibility Among Undergraduate Engineering Students: Findings from a Longitudinal Study

**DOI:** 10.1007/s11948-024-00462-8

**Published:** 2024-02-12

**Authors:** Shiloh James Howland, Brent K. Jesiek, Stephanie Claussen, Carla B. Zoltowski

**Affiliations:** 1 Educational Inquiry, Measurement, and Evaluation, Brigham Young University, Provo, USA; 2 School of Engineering Education, Purdue University, West Lafayette, USA; 3 School of Engineering, San Francisco State University, San Francisco, USA; 4 School of Electrical and Computer Engineering, Purdue University, West Lafayette, USA

**Keywords:** Longitudinal, Survey, Ethics, Social responsibility, Education

## Abstract

Prior research on engineering students’ understandings of ethics and social responsibility has produced mixed and sometimes conflicting results. Seeking greater clarity in this area of investigation, we conducted an exploratory, longitudinal study at four universities in the United States to better understand how engineering undergraduate students perceive ethics and social responsibility and how those perceptions change over time. Undergraduate engineering students at four U.S. universities were surveyed three times: during their 1st (Fall 2015), 5th (Fall 2017), and 8th semesters (Spring 2019). The students who completed all three surveys (*n* = 226) comprise the sample that was analyzed in this paper for changes in their scores on five instruments: Fundamentals of Engineering/Situational Judgment, Moral Disengagement, ABET Engineering Work and Practice Considerations, Macroethics, and Political and Social Involvement Scale. We found that students modestly increased their knowledge of ethics and ability to apply that knowledge in situations calling for them to exercise judgment. In addition, they consistently indicated that health and safety considerations in engineering were of highest importance. They also showed steady levels of social consciousness over time, in contrast to other studies which detected a culture of increasing disengagement in engineering students throughout the four years of their undergraduate studies.

## Introduction

Many reports and policy documents have underscored the need to cultivate social and ethical responsibilities among current and future engineers (National Academy of Engineering, 2004; Sheppard et al., 2009). Additionally, ABET’s most recent guidelines for accrediting engineering degree programs single out ethics as one of seven required learning outcomes (Criterion 3.4), specifically calling for graduates to have: “an ability to recognize ethical and professional responsibilities in engineering situations and make informed judgments, which must consider the impact of engineering solutions in global, economic, environmental, and societal contexts” (ABET 2018, p. 5). The third edition of the American Society of Civil Engineers’ Civil Engineering Body of Knowledge Report (American Society of Civil Engineers 2019) likewise identifies ethics as a learning outcome separate from professionalism, a move designed “to specifically emphasize ethics because of its importance to the individual civil engineer and the civil engineering profession” (p. 150).

Nonetheless, an established but still growing body of anecdotal and empirical evidence suggests a persistent lack of serious attention to ethics, social responsibility, and allied topics in most engineering degree programs (e.g., see Stephan, 1999; Colby & Sullivan, 2008; Hess & Fore, 2018). As Hess and Fore summarize in the most recent systematic review on engineering ethics interventions, U.S. engineering programs at many schools lack “an explicit focus on students’ ethical development” (2018, p. 552). Their review additionally reveals wide variation in how ethics is treated in existing engineering courses and curricula, including how many and what kinds of topics are covered, as well as different pedagogical approaches (Hess & Fore, 2018).

To build stronger evidence-based foundations for growing and improving ethics education in engineering, our research team carried out a longitudinal, mixed-methods study of undergraduate engineering students at four U.S. universities. In this paper we specifically report on a subset of our data, namely a set of repeat survey measures collected from undergraduate engineering students during the 1st, 5th, and 8th semesters of their studies. Our analysis of this data is guided by one research question:

How do foundational measures and understandings of social and ethical responsibility change during a four-year engineering degree program?

By “foundational measures,” we refer to a variety of survey items and instruments, some of which have been used and reported on elsewhere in the literature, that aim to measure various facets of social and ethical responsibility in engineering. Our attention to “social and ethical responsibility” is broadly construed within the context of engineering education and professional practice. It includes perceptions of engineering ethics and professional integrity as reflected in professional codes of ethics (e.g., National Society of Professional Engineers, 2019), ABET accreditation criteria, and the Professional Engineer (PE) licensure process, as well as larger questions of collective social responsibility and “macroethics,” including social justice and other considerations (e.g., Herkert, 2005; Leydens et al., 2012; Riley, 2008).

Collectively, the measures we selected were chosen to cover a broad swath of social and ethical responsibility constructs. This research was designed to be exploratory rather than explanatory with the hope that our findings would point future researchers to avenues worthy of further investigation. As we write now, we have the benefit of almost 10 years of hindsight from when the project was first conceived in 2014. Our goal was to measure change over time (if it existed) and in doing so, we could not change the measures we selected after the initial survey, even if new measures became available or if early results suggested that a construct we thought was worth investigating turned out to be less fruitful than we initially hoped.

This paper addresses the preceding research question by reporting on students’ responses to a variety of quantitative measures over four years. The paper begins with a literature review followed by methods, findings, and discussion sections. We conclude with some possible implications for a variety of audiences, including researchers, instructors, and administrators who develop, deliver, or oversee ethics interventions in engineering.

## Literature Review

Social and ethical responsibility have been described in the literature in a wide variety of ways, informed by general frameworks drawn from other fields (e.g., philosophy, psychology, sociology) and specific professional concerns (i.e., in the context of engineering). Despite these variations, many studies have used a limited range of quantitative and qualitative measures to investigate how engineering students understand social and ethical responsibility.

Many studies exploring the impact of pedagogy on students’ understanding of ethical issues have used models of ethical development informed by frameworks drawn from moral psychology (Rest, 1982; Tuana, 2014) which consist of multiple components or elements (e.g., ethical sensitivity, ethical judgment or decision-making skills, ethical motivation, etc.). However, researchers have often used more narrow measures to assess students’ understandings of these multi-faceted concepts, with a focus primarily on ethical decision-making skills using surveys such as the Defining Issues Test, Version 2 (DIT-2) (Rest et al., 1999) or the Engineering Ethical Reasoning Instrument (EERI) (Zhu et al., 2014a), with mixed results. For example, a study by Loui (2006) reported modest increases in DIT-2 scores after engineering students were exposed to a video-based case study of an engineering ethics dilemma, whereas Wu et al. (2008) found no significant differences when using the DIT-2 to assess moral development of engineering students at one U.S. university across classes ranging from first-year students to seniors. Similarly, Drake et al. (2005) found that neither a full semester ethics course nor an ethics module in an engineering course resulted in significant improvements in DIT-2 scores. A study by Hess et al. (2019) investigated the impact of integrating reflexive principlism, an ethical reasoning approach, into a graduate engineering class using multiple measures including the EERI and the DIT-2. The results were also mixed: the EERI indicated an increase in the ethical reasoning abilities of the students, but the DIT-2 did not. While not the focus of this paper, qualitative measures of ethical understanding have resulted in similarly mixed findings (Clancy et al., 2005; Feister et al., 2014a; Loui, 2005; Shuman et al., 2004).

Another scale, the Engineering and Science Issues Test (ESIT; Borenstein et al., 2010) is modeled on the DIT-2 test but with a narrower focus on the “technical dilemmas in science and engineering” (p. 387). Research using this instrument showed that junior and senior engineering students in certain ethics-related courses showed improvements in moral reasoning as measured by pre- and post-test ESIT scores. Yet as in other research, Borenstein et al. (2010) results were somewhat mixed. Some ethics-related courses had no measurable impact on moral reasoning—a finding that they note warrants further research as “some course curricula may be better than others at improving moral reasoning” (p. 405).

One of the largest mixed-methods studies of ethics in engineering education also produced varied results. Finelli et al. (2012) collected survey data from a large sample of engineering undergraduates at 18 U.S. institutions. The survey consisted of the DIT-2 plus a 152-item Student Engineering Ethical Development (SEED) survey to investigate three constructs of ethical development (knowledge of ethics, ethical reasoning, and ethical behavior) and various aspects of ethics-related experiences (types of experiences they had, their satisfaction with those experiences, and perceived importance of the experiences). Of the initial 3914 students, 450 students completed the survey again two years later (Harding et al., 2013). The research team found that students appeared to engage in higher levels of ethical reasoning as measured by the DIT-2 scores at the second time point but did not make meaningful gains in their knowledge of engineering ethics. They also documented increases in student participation in community-based projects and pro-social behavior (e.g., volunteering), but this was paradoxically paralleled by evidence of an increased likelihood of cheating among respondents (Harding et al., 2013).

Still other studies have more broadly looked at social responsibility and related commitments among engineering students. For instance, one early and influential study by Astin (1993) found that engineering graduates were more likely to have pessimistic views about their ability to change society and cultivate a meaningful philosophy of life as compared to individuals in other fields. Along similar lines, Sax et al. (2000) reported that engineering students in the U.S. had, on average, lower commitments to social action as compared to their peers in other disciplines. Multiple studies have additionally found connections between academic and workplace dishonesty, both in engineering (Harding et al., 2013) and business (Graves, 2008) contexts.

Canney and Bielefeldt (2015a) have more specifically conceptualized social responsibility as “feelings of obligation to help others as both a person and a professional, with a special focus on helping disadvantaged or marginalized populations” (p. 415). To assess this construct, they developed the Professional Social Responsibility Development Model (PSRDM) and the related Engineering Professional Responsibility Assessment (EPRA) (Bielefeldt & Canney, 2016). Using the EPRA across five U.S. institutions, they investigated civil, environmental, and mechanical engineering students’ social responsibility attitudes at the beginning (*n* = 1000) and end (*n* = 698) of an academic year. They report many intriguing results, including that environmental engineering students had more positive social responsibility attitudes than civil engineering students, who had more positive attitudes than mechanical engineering students, with 1st-year students in those majors showed the greatest differences (Canney & Bielefeldt, 2015b). In addition, they found that women had overall higher degrees of social responsibility than men, but those differences were generally lower for senior and graduate students (Canney & Bielefeldt, 2015c).

The extant literature has also explored how educational contexts impact the ethical development of engineering students. For example, Rudnicka (2009) has shown how engineering students’ ethical reasoning and decision-making are affected by “contextual/environmental factors” such as team learning dynamics, work experience, the culture of the engineering field, and the moral intensity of a given dilemma or situation. Studies by Feister et al. (2014b) and Zhu et al. (2014b) additionally found evidence that students working in teams conceptualized ethics and made ethical decisions differently based in part on programmatic orientations (e.g., entrepreneurship, business, or community engagement).

Perhaps one of the most influential papers in engineering ethics education in the past decade has been Cech’s (2014) work on the “culture of disengagement” in engineering education. In her longitudinal study of undergraduate engineering students at four Massachusetts universities (*n* = 326), results indicated that student interest in public welfare considerations tended to decrease over the four years they were enrolled in engineering programs. This alarming finding prompted concern that engineering students are being influenced by implicit and explicit messaging that “prioritize[s] the technical over the non-technical, including concerns about social and environmental implications, ethics, values, and meaning” (Snieder & Zhu, 2020, p. 2244).

Within the aforementioned studies which looked at changes in aspects of social or ethical responsibility with respect to specific learning environments and experiences, it is worth emphasizing that the results were mixed, especially for different types of measures (quantitative and qualitative). The seemingly contradictory results may suggest the complexity of the issues themselves, as well as the difficulty of detecting changes using one or a few measures focused on relatively narrow constructs. The body of prior work reviewed here suggests the need for more research, including studies that use multiple measures to explore longitudinal changes in perceptions and understandings of social and ethical responsibility.

## Methods

**Fig. 1:**
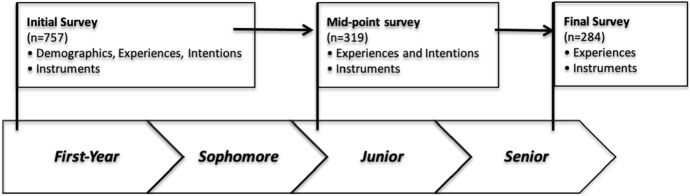
Data collection plan for project

The results presented in this paper come from three stages of quantitative data collection in a four-year longitudinal mixed-methods study of engineering students (shown in Fig. 1 as initial survey, mid-point survey, and final survey). Students were eligible to complete the initial survey in our study if they were age 18 years or older, true first-semester first-year students (i.e., not transfer students), and enrolled full-time in an engineering or technology major at one of four participating schools: (a public research-intensive and project-based university; ASU), Brigham Young University (a private, religious, research-intensive university; BYU), Colorado School of Mines (a public undergraduate-serving university; Mines), and Purdue University (a public, research-intensive university). While these universities may not be entirely representative of all U.S. universities, they were chosen to represent a variety of institution types (public and private, varying sizes, geographic regions, levels of research activity, etc.). All data collection was carried out under approved IRB procedures at each school.

### Longitudinal Data Collection

In the early fall (September–October) of 2015, 757 students responded to the initial survey in this study (results of this early work are found in Zoltowski, Jesiek, Claussen & Torres, 2016). These respondents were then contacted again to complete the mid-point survey in the fall of their junior year (September–October 2017) (results of this survey are found in Howland, Warnick, Zoltowski, Jesiek, & Davies, 2018). The final survey was distributed to the original respondents at the end of their senior year (January–February 2019), assuming a typical four-year degree progression. Students were eligible to participate in this final survey if they completed at least 6 semesters of study and were still enrolled in an engineering or technology major at one of the four collaborating universities. 226 students responded to all three surveys and provide the basis for the analyses presented here.

### Survey Measures

The survey was comprised of eight sections, each measuring an aspect of the students’ perceptions of ethics and social responsibility: Fundamentals of Engineering/Situational Judgment, Ethical Climate Index, Justice Beliefs, Political and Social Involvement Scale, Macroethics, Engineering Work and Practice Considerations, Moral Attentiveness, and Moral Disengagement. Where possible, we adopted measures with previously published results that exhibited acceptable evidence of validity and reliability with the intent of studying changes in their responses over time. In addition, we aimed to use foundational measures of social and ethical responsibility—measures that were used frequently elsewhere in the engineering education literature and broader literature on ethics. Thus, our survey allowed us to make meaningful comparisons both across the measures themselves and with findings from other research studies.

Median completion time for each survey was 25–30 min. Students who completed the surveys received $5 gift cards for the initial survey and $10 for the follow-up surveys. The instruments used in this study were selected to measure a wide variety of complementary constructs and measurement domains and include both general and engineering-specific measures. This paper reports on the changes seen in the students who responded to all three surveys (n = 226) on five instruments (Fundamentals of Engineering/Situational Judgment, Moral Disengagement, Engineering Work and Practice Considerations, Macroethics, and Political and Social Involvement Scale; a total of 55 items) over the four years of their undergraduate careers. These five instruments measure students’ ability to act in situations calling for judgment, interest in being involved in their communities, and their concerns about the role of engineers in serving their employers and the public. Some of our previous work has addressed changes on other instruments and analysis of results is ongoing (Fuentes et al., 2016; Howland et al., 2018; Jesiek et al., 2022; Nittala et al., 2019; Kim et al., 2018; Kim, Jesiek & Howland, 2021; Zoltowski et al., 2020; Stepback et al., 2023; Claussen et al., n.d). The sections that follow offer additional details about the five survey instruments reported on in this paper. A complete list of all items used in the survey is available in Appendix A.

### Fundamentals of Engineering and Situational Judgment

The Fundamentals of Engineering and Situational Judgment (FESJ) measure is comprised of eight multiple-choice items that assess students' knowledge of ethics and how to approach ethical dilemmas. A subset of five items present questions similar to those that appear on the Fundamentals of Engineering exam and have been used in previous studies of engineering ethics (Harding et al., 2013; Carpenter et al., 2015). Three additional items required respondents to indicate what actions they would take in a variety of ethical situations. These situational judgment scenarios were adapted from a previous project (Jesiek, Buswell, & Zhu, 2018) and were reviewed by multiple subject matter experts to help establish their validity. Items were scored as either correct (most desirable answer) or incorrect (for the other, less desirable answers). Responses to this measure were analyzed using repeated-measures ANOVA, followed by pairwise comparisons with a Bonferroni adjustment to correct for multiple comparisons,

### Moral Disengagement Scale

The Moral Disengagement scale is composed of 24 items that measure students’ propensity to engage in unethical behaviors, with a focus on eight specific cognitive mechanisms (each measured using three items) that may lead individuals to disengage or disregard the morality of their actions (Detert et al., 2008). For example, the item, “It is alright to fight to protect your friends” reflects the disengagement mechanism called “moral justification.” These items were measured on a five-point Likert scale from *strongly disagree* to *strongly agree*. Though we are unaware of this scale being used in other engineering education research, we believe that its 24 items adequately captured a variety of mechanisms of moral disengagement, each of which was worth investigating.

### Engineering Work and Practice Considerations

Students were asked to rate the importance of the seven ABET considerations of engineering practice (technical, social, economic, manufacturability, environmental, health and safety, and ethical, ABET 2018) on a four-point scale (*not at all important* to *extremely important*). These items were previously used in a study of engineering students’ development from students to professionals (Huff, 2014) and are drawn directly from ABET student outcome statements. All analyses included a Bonferroni adjustment. When conducting multiple analyses of the same dataset, a Bonferroni adjustment reduces the alpha value (generally 0.05) to compensate for the additional analyses. A Bonferroni adjustment divides the alpha value (0.05) by the number of analyses (three pairwise comparisons in this instance—initial to midpoint, midpoint to final, and initial to final). This reduces the chance that a statistically significant result is found just due to the number of analyses.

### Macroethics

Students rated their level of agreement with four statements, detailed below, about the macroethical concerns of engineers using a five-point scale (*strongly disagree* to *strongly agree*). These items are not previously published, but were adapted from previous research efforts (Ellison et al., 2013) and from various engineering codes of ethics as they have evolved over time. The responses to these four statements were analyzed using non-parametric related-samples Friedman’s two-way analysis of ranks to look for differences over time within individual student responses. If that test showed a statistically significant result, pairwise comparisons were examined using a Bonferroni adjustment for multiple analyses to identify when those differences occurred.

### Political and Social Involvement Scale

The Political and Social Involvement Scale (PSIS) asked students to rank the personal importance of twelve social and political activities (e.g., *Enhancing racial understanding*) on a four-point Likert scale. Similar items were used in the Wabash National Study of Liberal Arts Education (Blaich & Wise, 2011; Pascarella, 2007). Four items of the items from this scale used in our study are very similar to four items deployed by Cech (2014) as a measure of what she referred to as “social consciousness,” which, as noted above, identified concerns about engineering students’ growing disengagement during their undergraduate studies. We conducted analyses using a Friedman test to determine if there were statistically significant changes in responses across time at the individual item level. This non-parametric statistical test was selected as it allows us to compare results from three timepoints for ordinal level data (*n* = 221). This analysis used the full four-point scale to measure these changes in importance over time.

### Demographic Information

Basic demographic information about the 226 students who responded to the three surveys is shown below (Table 1). Brigham Young University (BYU) had a higher rate of attrition due to the fact that many students at BYU complete an 18 or 24 month volunteer church service mission while at the university, precluding many students from meeting the eligibility requirements to complete the mid-point (2017) and final (2019) surveys. Recruiting efforts at Arizona State University were hampered by difficulties in accessing engineering majors as they were spread across several campuses and by turnover in the members of our research team from that school.

**Table 1:** Number of responses to each survey

University	2015 initial survey	2017 mid-point survey	2019 final survey	Responded to all 3 surveys
Arizona State University	86	32	21	16
Brigham Young University	209	44	43	33
Colorado School of Mines	218	129	115	96
Purdue University	244	114	105	81
Total	757	319	284	226

Demographic details are provided for the students who completed all three surveys (*n* = 226, Table 2). Compared to national averages, our sample was over-represented in some categories. Our sample was 34.5% female compared to the national average of 23.8% of enrolled engineering students who are female (American Society for Engineering Education 2020). Only 4.8% of respondents in our sample were international students, compared to 9.4% nationally (American Society for Engineering Education 2020).

**Table 2:** Demographic information students who completed all three surveys (n = 226)

University	Student status		Gender		Ethnicity	
	International	Domestic	Male	Female	White	Non-white
ASU	1	15	12	4	6	10
BYU	1	32	27	6	29	4
Mines	0	96	64	32	76	20
Purdue	9	72	45	36	58	23
Total	11	215	148	78	169	57

### Data Analysis

Prior to data analysis, students’ responses to the 55 items on the final survey were matched to their responses on the two previous surveys. Statistical analysis for some instruments was conducted using repeated-measures ANOVA, while other forms of analyses were used for some instruments when it was permitted by the data or to make our results more comparable to previous work. Repeated-measures ANOVA allows comparisons of matched data (within-individual) change over multiple time points. The findings presented below provide additional details about the specific analytic techniques used in this study, in alignment with the characteristics of our data set and the individual measures.

In order to establish whether there were differences between the students who completed all three surveys and the students who did not, independent sample *t*-tests were used to compare the mean scores of the five instruments using the initial survey data from 2015. For each of the five instruments, there were no statistical differences in the means of the scores on the instruments for students who completed all three surveys (*n* = 226) compared to those students who completed only one or two surveys (*n* = 531).

## Results

In each of the following sections, we generally only discuss statistically significant results. We again want to emphasize the exploratory nature of this research. Our intent is to report what we found from our subjects and our measures, with the hope that our results may help future researchers focus more directly on promising measures that can adequately capture the changes and lack of changes in engineering students’ development of ethical and social responsibility over time.

### Fundamentals of Engineering and Situational Judgment

Repeated-measures ANOVA showed that students’ average FESJ scores (*n* = 220) increased by a statistically significant amount (*F*(2,438) = 4.563, *p* = 0.011, partial eta squared = 0.020) between the initial and final surveys. There was no statistically significant change in average scores between the initial and mid-point or the mid-point and final surveys (Table 3).

**Table 3:** Changes in FESJ scores over time

		Difference (*p*-value)	
Survey	Average FESJ score (standard deviation)	Compared to initial survey (2015)	Compared to mid-point survey (2017)
Initial (2015)	5.57 (1.5)	Not applicable	Not applicable
Mid-point (2017)	5.82 (1.4)	0.25 (0.097)	Not applicable
Final (2019)	5.91 (1.5)	0.34 (0.018)*	0.13 (1.000)

^*^Statistically significant difference

From the changes in their responses to the Fundamentals of Engineering/Situational Judgment questions, we can conclude that this particular group of students improved in their knowledge of how to respond to these situations. On average, the respondents correctly answered 5.6 questions (out of eight) as first semester students and they improved their scores on this measure over time, rising to an average score of 5.9 correct answers. No differences were seen on this measure based on gender or university.

### Moral Disengagement

Our analysis detected no differences in overall scores on this instrument over the three surveys, as determined by a repeated-measures ANOVA (*n* = 217; *F*(2,432) = 1.190, *p* = 0.305; Table 4). From this result, we infer that the students in our study did not become more prone to morally disengage across the 4 years we studied them. This finding, as will be discussed further below, may run counter to other past work which identified a “culture of disengagement” among engineering students. Cronbach’s alpha for this scale was calculated for each time point and found to indicate acceptable levels of internal consistency: 0.895 (2015), 0.751 (2017), and 0.757 (2019).

**Table 4:** Moral disengagement scores over time

Survey	Scores (standard deviation)
Initial (2015)	48.7 (9.7)
Mid-point (2017)	47.7 (10.4)
Final (2019)	48.4 (9.9)

### Engineering Work and Practice Considerations

Of the seven considerations presented to students, Health & Safety was consistently rated highest, on average, followed by Technical and Ethical considerations. Social considerations were rated lowest in all three surveys (Table 5).

**Table 5:** Relative rankings of ABET considerations

Consideration	Initial survey	Mid-point survey	Final survey
Health & Safety	1	1	1
Technical	2	2	2
Ethical	3	3	3
Manufacturability	4	5	6
Economic	5	6	5
Environment	6	4	4
Social	7	7	7

1 = highest importance, 7 = lowest importance, *n* = 221

Because these data are strictly ordinal, related-samples Friedman’s two-way analysis of ranks was used to look for differences in the distribution of student responses over time. Two considerations showed changes over time—environment and manufacturability. Students’ ranking of the importance of environmental concerns changed over time (χ^2^ = 12.081, *df* = 2, *p* = 0.002). Pairwise analysis showed that *environmental* concerns were more important on the final survey compared to the initial survey (*p* = 0.013). Also, students’ ranking of the importance of manufacturability concerns changed over time (χ^2^ = 11.698, *df* = 2, *p* = 0.003). Specifically, pairwise analysis showed that *manufacturability* was perceived as less important on the final survey compared to the first survey (*p* = 0.010). These findings, including the perception of social considerations being less important than other considerations, are discussed below. Nonetheless, we can see from these results that undergraduate engineering students uniformly ranked health and safety of highest importance over time.

### Macroethics

Two statements showed no statistically significant changes in the distribution of student responses over time: “Scientists and engineers ought to educate the public about options and implications of innovations so that people can meaningfully promote, regulate, or otherwise engage with new technologies” and “An engineer’s first duty is to the public.”

For the statement “Surprising and risky uses of new technologies, such as social networking websites, are completely the responsibility of people who use them,” students’ responses changed over time (χ^2^ = 68.351, *df* = 2, *p* < 0.001). More students indicated on the final survey that they disagreed with this statement compared to their responses on both the initial survey (*p* < 0.001) and the mid-point survey (*p* < 0.001). Additionally, for the statement, “An engineer’s first duty is to his or her employer,” students’ responses changed over time (χ^2^ = 23.598, *df* = 2, *p* < 0.001). On the final survey, more students indicated that they disagreed with this statement compared to the first survey (*p* < 0.001). The changes in student responses to these Macroethics items suggest that students are maintaining or increasing their level of concern for the public over time, as we will discuss further in the Discussion.

### Political and Social Involvement Scale

Overall, there was no difference across time in the number of activities on this scale that students rated as *essential* or *very important*. At each time point, the students selected 6.8 of the twelve activities as *essential* or *very important*.

Though the students overall held 6.8 of the 12 activities to be consistently *very important* or *essential*, there could be offsetting changes where they held one activity to be less important over time and another activity to be more important over time. Statistically significant decreases were found for two of the activities:

Becoming a community leader – This activity *decreased* in its importance to participants over time (χ^2^ = 11.162, *df* = 2, *p* = 0.004).

Integrating spirituality into my life – This activity *decreased* in its importance to participants over time (χ^2^ = 6.225, *df* = 2, *p* = 0.011).

Additionally, statistically significant increases were found for two of the activities:

Keeping up to date with political affairs – This activity *increased* in its importance to participants over time (χ^2^ = 9.363, *df* = 2, *p* = 0.009).

Helping to promote racial understanding – This activity *increased* in its importance to participants over time (χ^2^ = 7.474, *df* = 2, *p* = 0.024).

This same analysis found no statistically significant differences between either the initial and mid-point surveys or between the initial and final surveys for how students rated the importance of the other eight items.

We also sought to replicate a part of Cech’s frequently cited longitudinal study which shows evidence of declining student interest in “public welfare concerns” during their undergraduate studies (Cech, 2014). In her study, Cech created a four-item “social consciousness scale,” finding a small but significant decrease in scores on this measure among engineering students (*n* = 326) from their 1st to 4th year of college. Cech’s items were: *helping others in need, promoting racial understanding, being active in my community,* and *improving society.* The four items from the PSIS we used were: *helping others who are in difficulty, helping to promote racial understanding, volunteering in my community,* and *improving society*. To be consistent with her study, we only used responses from the initial survey and the final survey (*n* = 278) and we averaged the students’ responses on a four-point Likert scale to create a social consciousness scale score (Cronbach’s alpha for 2015 data = 0.710, Cronbach’s alpha for 2019 data = 0.707; Table 6). The students in our study show no statistically significant change over time on this scale (*p* = 0.560).

**Table 6:** Comparison of social consciousness scores

Timepoint	Cech, 2014 (n = 326)		This study (n = 278)	
	Mean (standard error)	95% confidence interval	Mean (standard error)	95% confidence interval
T1 (first year)	2.940 (0.038)	2.866–3.014	2.971 (0.036)	2.901–3.042
T2 (senior year)	2.737 (0.049)	2.641–2.833	2.952 (0.036)	2.882–3.022

These were calculated using the means and standard errors Cech provided as well as the given sample size of 326

## Discussion

This paper investigates how engineering students’ perceptions of ethics and social responsibility changed over the course of 4 years of undergraduate study. In summary, we see that students slightly improved in their ability to respond to situations calling for knowledge and judgment and did not show evidence of an increase in disengagement with public welfare concerns over the 4 years they were surveyed (as measured by their responses to the Moral Disengagement, Engineering Work and Practice Considerations, Macroethics, and PSIS instruments). These findings offer some hope that engineering students are at least maintaining concern for public welfare over their undergraduate careers. We now turn to a more detailed discussion of our results in relation to other research on students’ understanding of ethics and social responsibility.

We begin with a discussion of the lack of overall changes on the PSIS. In contrast to Cech’s (2014) social consciousness scale results, students in our study showed consistent concern for public welfare throughout the 4 years they participated. Results from our study on the PSIS scale—combined with the increase in the respondents’ scores on the Fundamentals of Engineering and Situational Judgment scale, steady Moral Disengagement scores, consistent concern for non-technical factors (as measured by the Engineering Work and Practice Considerations items), and changes on the Macroethics items that imply a stronger identification with the public rather than employers (all further discussed below)—leads us to conclude that the students in our study have at least stable levels of awareness for their social and ethical responsibilities as aspiring engineers.

Additionally, responses to our PSIS items indicate no significant changes overall, although we did find a significant decrease for two items (*Becoming a community leader* and *Integrating spirituality into my life*) and significant increases for two items (*Keeping up to date with political affairs* and *Helping to promote racial understanding*). Though we hesitate to speculate why two items declined in importance, we note that the study period included the 2016 presidential election year in the United States and paralleled intensified national discussions around issues of race and social justice. This may help explain why many students reported a greater orientation toward the two items noted above during the particular period of this study.

Looking at individual items on the PSIS, some items decreased in relative importance to our respondents over time while others increased in importance. This is apparently contradictory, i.e., to state in one instance that a particular individual item increased in importance over time and to simultaneously say that we detected no change over time in the overall social consciousness scale. We believe this to be a matter of the different analyses used for the individual PSIS items and the social consciousness scale. Our analysis of each PSIS item, which showed some decreases and increases in four items, extended across all three timepoints whereas the social consciousness scale we created only examined a set of four PSIS items and used two timepoints (to be in line with Cech, 2014).

Regarding the Fundamentals of Engineering and Situational Judgment measure, our findings somewhat diverge from prior research. In a survey administered to a large, stratified sample of U.S. engineering students (n = 3,914) using five of the same questions, respondents answered about 3 of 5 items correct (or 60%), on average (Finelli et al., 2012). A follow-up study by Harding et al. (2013) additionally found no significant performance changes on this same measure among 450 students polled initially and 2 years later. In contrast, average performance on our similar eight-item measure was comparatively higher to begin with (5.57 out of 8 items, or about 70% correct), followed by a small increase after the 4 years of our study (rising to 5.91 out of 8 items in Year 4, or about 74%, for the final survey). While the overall higher scores in our study could be due to differences in measurement approaches, sample characteristics, or other factors, the longer overall duration of our study may help explain our encouraging result that performance on this measure increased modestly but significantly over time. That increase, and the fact that the respondents in this study started with relatively high scores, suggests that students have considerable knowledge or intuition around engineering ethics topics and issues even in their 1st year of their undergraduate studies. These findings suggest that current engineering ethics education efforts are having an impact, albeit a small one, but that additional instruction could yield further improvements in students’ ability to answer knowledge- and situation-based ethics questions. Our findings also suggest the possible need for more sensitive measures of engineering ethics knowledge.

Regarding moral disengagement, we saw that students’ responses to this scale did not change over time. The lack of change in overall scores for Moral Disengagement is consistent with previous work completed earlier in this longitudinal study (Howland et al., 2018). However, there were changes on three of the eight subscales within the Moral Disengagement scale (scores on *displacement of responsibility* increased over time while scores on the subscales *diffusion of responsibility* and *attribution of blame* decreased over time, as reported in Kim, Jesiek, & Howland, 2021). For this instrument, an increase in scores on a subscale indicates increased moral disengagement. Given these somewhat contradictory results, more research is needed to explore moral disengagement’s relevance to engineering education and practice, including to develop interventions that help students become more aware of how specific mechanisms of disengagement (*e.g.*, displacement of responsibility) may be linked to unethical behaviors.

Turning to the Engineering Work and Practice Considerations items, across all three surveys students consistently ranked health and safety, technical, and ethical considerations as respectively most important for engineering work, while environmental factors increased from the 6th to 4th most important factor. The fact that so many students repeatedly ranked health and safety as their top concern, and became more concerned about environmental considerations, is heartening, especially when compared to Cech’s finding where she reported that students tended to rate “technical” factors (*e.g.*, background in math and science, innovation, advancement of scientific knowledge) as relatively more important than other types of considerations (Cech, 2014). The falling rank of manufacturability in our data, on the other hand, may be a consequence of displacement as other considerations are perceived as relatively more important, or due to a decreasing sense of relevance as students move into specific fields and specialties where manufacturing is viewed as a less central concern. It is perhaps also disconcerting that social considerations were ranked lowest in all instances of the survey. However, the rather broad, vague nature of this one item may have caused it to be less understood and, therefore, ranked lower compared to other options. Yet the increase in consideration for environmental factors, along with consistently high rankings for health and safety and ethical considerations, again suggests that the students in this sample have persistent concern and appreciation for non-technical aspects of engineering.

Results for the Macroethics questions were also unexpected in relation to another set of Cech’s results. Her work found significant decreases over time in student perceptions of the importance of professional/ethical responsibilities and understanding the consequences of technology (Cech, 2014). By contrast, our findings indicated that students held consistent views about the role of engineers in educating the public and the duty of engineers to serve the public. And because more students disagreed over time with statements about engineers’ allegiance to their employers and the responsibility of users in relation to new technologies, we propose that these results suggest rising concern among engineering students regarding their responsibilities to the public and profession as they approach graduation.

We make three conclusions from our research: (1) Students in our study entered with, and maintained, an awareness of ethical and social issues. (2) Engineering ethics education efforts are helping to have a positive impact and are able to counterbalance a primary focus on technical aspects of engineering. (3) Current events may influence students’ perceptions of some aspects of social considerations. Nonetheless, there remain open questions about whether these commitments are adequate, including in relation to curricular objectives (e.g., ABET Criterion 3.4) that include ethics-related learning outcomes for engineering graduates. As noted below, there is also the question of whether and how such commitments change as students transition from school to work.

## Limitations

One significant limitation of this study is that the students were not randomly selected either from the four universities (the participants volunteered to participate in the study) or from the larger population of undergraduate engineering students throughout the United States. This limits the external validity of these results because they should not be generalized to a larger population. Additionally, there may be unique characteristics of students who elect to participate in a survey about ethics and social responsibility as compared to students who do not, which also could limit the generalizability of these results. And while the initial survey data was also re-analyzed to see if there were differences between students who only completed one or two surveys compared to the students who completed all surveys and no significant differences were found, it is possible there are other differences between these groups that were not identified.

Additionally, the measures we selected represent only some facets of student perceptions of social and ethical responsibility. This was necessary given the limited number of relevant, published measures with evidence of their validity. Some available measures were too lengthy to include (notably, the DIT-2, SEED, ESIT, and EERI scales are quite long and focus on narrow aspects of ethics and social responsibility) so the instruments here were selected to allow us to efficiently measure several related constructs. An additional measurement challenge arose from the fact that the constructs explored here and in other work are often not well-defined or have varying definitions between disciplines. More work is needed to collect additional evidence about the validity of inferences that can be made from using these and other measures of social and ethical responsibility.

Additionally, our study did not include a comparison group of non-engineering students. As such, we can only situate our findings within the realm of engineering students at these four U.S. universities and cannot say if such change or lack of change is similar to or different from undergraduate students in other fields of study. Future research that builds on these findings or measures would benefit from the inclusion of comparison groups.

## Conclusion

Findings from this study point toward some tentative implications and directions for future research. Cech (2014) has argued that the disciplinary culture of engineering at many schools tends to erode public welfare concerns among engineering students, reducing their concern with the broader impacts of engineering work as they progress toward a degree. We were not able to replicate Cech’s findings, instead observing no major changes, and in some cases even modest improvements, on a number of measures related to students’ ethical understanding and concerns. We are heartened to see that a culture of disengagement feared to be present in engineering schools may not be as pervasive as we once thought. This is admittedly a study with a limited scope (as was Cech’s) but our results may be evidence that students are at least able to maintain concern for the public’s welfare throughout their undergraduate careers. As stated earlier, it is our hope that other engineering education researchers can use our exploratory findings as a starting point for their own attempts to understand how engineering students’ perceptions of ethics and social responsibility develop during their time as undergraduate students.

Yet we also did not find evidence of particularly strong commitments toward public welfare and social responsibility among our subjects. Of course, this begs the question of what specific evidence is needed to make robust claims about engineering students tending, on the whole, toward greater social disengagement. More research is needed to establish baseline data for comparing such outcomes and constructs, including across different disciplinary areas, levels of school, and career settings and comparing between engineering and non-engineering students. Further, Cech found stable or even slight declines in public welfare beliefs among the subjects in her study when they went on to full time work (Cech, 2014). We are currently carrying out a follow-on study where we are collecting similar survey data from the same group of survey respondents profiled in this paper now that most have completed their undergraduate education and either entered the workforce or started graduate studies. We look forward to reporting more about these students as they transition to the workforce.

Another major aspect of research in our larger study centers on the qualitative (interview) data we collected from a subset of our survey respondents during their first and final years of schooling. We are now exploring whether this data confirms or refutes some of the key findings presented above (e.g. in Claussen et al., n.d.). We hope that the insights gleaned from all the strands of our project will help the larger engineering education community better understand, and positively impact, the social and ethical commitments of engineering students and professionals.

## Data Availability

Access to de-identified project data will be provided through a direct request to the project PI (Brent K. Jesiek, email address: bjesiek@purdue.edu)

## Appendix A: Survey Instruments

### Fundamentals of Engineering/Situational Judgment (FESJ)

1. What is the best word to complete the following sentence: Engineers are to uphold the health, safety, and ____________ of the public.
  A. trust
  B. infrastructure
  C. confidence
  D. welfare (correct)
2. Jean, a consulting civil engineer, has been hired by a multinational paper products conglomerate to provide both professional analyses of the enterprise's new plant design, as well as representation before the county compliance commission governing the proposed site. After conducting on-site soil analysis, Jean discovers that a chemical seepage into the local aquifer, though a low probability, would likely cause a much higher degree of water contamination than earlier estimates predicted. Upon contacting her client, expressing her concern with the new data, and her opinion that both be shared with the county commission immediately, the conglomerate executive officer responsible for the project informs her that the new data is irrelevant and should not be mentioned in Jean's upcoming public presentation before the commission.
  Of the following, which would be Jean's best action?
  A. Share her new findings at the public presentation.
  B. Terminate her consulting contract with the conglomerate. (correct)
  C. Contact the conglomerate's board of directors, express her disagreement with the executive officer's opinion, and make the case for sharing the new data.
  D. Follow the executive officer's lead and do not share the new data with the commission.
3. Muriel, a consulting computer engineer, is hired to review her client's plans to expand its existing computer network. In the course of completing her examination of the client's internal communication capacities and requirements, Muriel discovers that the client is using an unlicensed version of a popular proprietary software package to manage its financial accounts. Since her finding is only indirectly related to her work, Muriel is not sure what she should do.
  Of the following options, which one should Muriel NOT choose?
  A. Since the unlicensed software is not directly related to her work, Muriel should ignore it and complete her assignment. (correct)
  B. Muriel should bring the matter to the attention of her client’s executive officer and terminate her contract.
  C. Muriel should refuse to continue her work for the client unless the unlicensed software is immediately replaced with a properly licensed version.
  D. Muriel should not allow the client to use her name in advertising materials.
4. When Andrew, a professional engineer, discovers evidence that leads him to strongly believe his supervising engineer is attempting to injure the reputation of a competing firm, what should Andrew do?
  A. Andrew should focus on doing his own work, not on criticizing others.
  B. Andrew should inform the NCEES (National Council of Examiners for Engineering and Surveying) Licensing Board of his evidence and assist it in determining the truth of the matter. (correct)
  C. Andrew should resign from his job.
  D. Andrew should speak to the supervising engineer in order to determine the rationale for his actions.
5. Langdon, a consulting electrical engineer, is hired by PixDream, a major motion picture company, to design and oversee the construction of the power distribution system at the company's new film studio. Once the system is in place, PixDream asks Langdon to accept a 9 month contract extension, and to monitor the power system during the filming of Monster Mountain. He accepts the contract extension. Three weeks into the shoot, with the power system operating well within acceptable parameters, Langdon is asked by PixDream to give his opinion on a pyrotechnic specialist's plan for detonating a series of explosive charges. The charges are triggered electrically, but their chemistry does not fall within Langdon's expertise. PixDream is confident Langdon can become familiar enough with the charges to give them a professional and competent opinion. Langdon wants to continue working for PixDream, but is uncomfortable with the idea of giving his professional opinion on matters beyond his area of expertise.
  Of the following, which is Langdon's best option?
  A. Since he enjoys the work, Langdon can learn about a charge's chemistry and give PixDream his opinion on the pyrotechnic specialist's plan.
  B. Langdon can trust that the pyrotechnics specialist is knowledgeable and trustworthy, and give PixDream a favorable assessment of the plan.
  C. Langdon should contact some of the specialist's previous clients and base his analysis on their degree of satisfaction or dissatisfaction with the specialist’s work.
  D. Langdon should decline to accept the contract extension on grounds that explosives chemistry is beyond both his engineering education and subsequent work experience. (correct)
6. As a software engineer working for a multinational information technology services provider, you are assigned to apply routine updates to a customized payroll system your firm developed for a client. While reviewing the code, you notice the application has been modified to keep two sets of records, likely to hide underpayment of wages and excessive overtime at the client firm. The client's contract allows them to change the software code, and their modifications will not interfere with your updates.
  What would you do in this situation?
  A. Covertly reverse the modifications.
  B. Report the issue to your supervisor. (correct)
  C. Ask your supervisor to assign you to a different project.
  D. Ignore the modifications and continue with your work.
7. As an engineer employed by a multinational company, you recently made some major contributions to solving a critical issue in the design of a new product. You and two of your colleagues were praised in private by your manager for your significant roles in solving the problem, and he also told you he would submit a technical paper about the innovation to a major journal. When your manager circulates a final draft of the paper, you notice that he is listed as the only author.
  What would you do in this situation?
  A. Request a one-on-one meeting with your manager to discuss the issue. (correct)
  B. Send the editor of the journal a confidential note describing your concerns.
  C. Raise your concerns during dinner and drinks with your manager and colleagues.
  D. Take no further action.
8. You are an electrical engineer working for a large, multinational telecommunications firm. In a recent meeting, your supervisor hands you a job description for a new network installation project and asks you to identify a suitable contractor to perform the work. Yet when you review your company's online contractor database, you find that the language in the job description your supervisor gave you is nearly identical to what appears in one of the contractor's profiles. The match seems a little too perfect, and you are unsure of your supervisor's motivations.
  What would you do in this situation?
  A. Recommend the second best contractor in the database, ignoring the closest match.
  B. Ask your supervisor to assign this task to someone else because you are too busy.
  C. Do as you are told, recommending the contractor that most closely matches the job description.
  D. Confront your supervisor about the similarities between the job description and the contractor’s profile. (correct)

### Political and Social Involvement Scale (PSIS)

Respondents selected from a four-point Likert scale (*not important*, *somewhat important*, *very important*, *essential*). The instructions were: “For each question below, mark the response that most closely indicates what you think or feel. There is neither a right nor wrong answer to any question.”

A. Becoming a community leader.
B. Becoming involved with activities that preserve and enrich the environment.
C. Helping others who are in difficulty.
D. Improving my understanding of other countries and cultures.
E. Keeping up to date with political affairs.
F. Developing a meaningful philosophy of life.
G. Helping to promote racial understanding.
H. Influencing social values.
I. Influencing the political structure.
J. Integrating spirituality into my life.
K. Volunteering in my community.
L. Improving society.

### Engineering Work and Practice Considerations

Respondents selected from a five-point Likert scale (*strongly disagree* to *strongly agree*). The instructions were: “As you think about the field of engineering generally, rate the importance of each of these considerations in relation to engineering work (or the practice of engineering).”

- Technical
- Environmental
- Social
- Economic
- Health & Safety
- Manufacturability
- Ethical

### Macroethics

Respondents selected from a five-point Likert scale (*strongly disagree* to *strongly agree*).

1. Scientists and engineers ought to educate the public about options and implications of innovations so that people can meaningfully promote, regulate, or otherwise engage with new technologies.
2. Surprising and risky uses or new technologies, such as social networking websites, are completely the responsibility of people who use them.
3. An engineer's first duty is to his or her employer.
4. An engineer's first duty is to the public.

### Moral Disengagement

Respondents selected from a five-point Likert scale (*strongly disagree* to *strongly agree*). The instructions were: “Indicate the extent to which you agree with each statement below.”

1. Indicate the extent to which you agree with each statement below.—It's alright to fight to protect your friends.
2. It's ok to steal to take care of your family's needs.
3. It's ok to attack someone who threatens your family's honor.
4. Sharing test questions is just a way of helping your friends.
5. Talking about people behind their backs is just part of the game.
6. Looking at a friend's homework without permission is just borrowing it.
7. Damaging some property is no big deal when you consider that others are beating up people.
8. Stealing some money is not too serious compared to those who steal a lot of money.
9. Compared to other illegal things people do, taking some things from a store without paying for them is not very serious.
10. If people are living under bad conditions, they cannot be blamed for behaving aggressively.
11. If someone is pressured into doing something, they shouldn't be blamed for it.
12. People cannot be blamed for misbehaving if their friends pressured them to do it.
13. A member of a group or team should not be blamed for the trouble the team caused.
14. If a group decides together to do something harmful, it is unfair to blame any one member of the group for it.
15. You can't blame a person who plays only a small part in the harm caused by a group.
16. People don't mind being teased because it shows interest in them.
17. Teasing someone does not really hurt them.
18. Insults don't really hurt anyone.
19. If someone leaves something lying around, it's their own fault if it gets stolen.
20. People who are mistreated have usually done things to deserve it.
21. People are not at fault for misbehaving at work if their managers mistreat them.
22. Some people deserve to be treated like animals.
23. It is ok to treat badly someone who behaved like a worm.
24. Someone who is obnoxious does not deserve to be treated like a human being.
